# Refractory ventricular tachycardia storm with 42 ICD shocks in 24 h: A case report

**DOI:** 10.3892/mi.2026.306

**Published:** 2026-03-03

**Authors:** Parth Adrejiya, Negarsadat Neshat, Abdel Rahman Qamar, Harsh Suthar, Ngozika Orjioke, Abhishek Thandra, Divyang Patel

**Affiliations:** 1 Department of Internal Medicine, Wellstar Spalding Medical Center, Griffin, GA 30224, USA; 2 Department of Internal Medicine, Wellstar Kennestone Medical Center, Marietta, GA 30060, USA; 3 Department of Internal Medicine, Smt N H L Municipal Medical College, Ahmedabad, Gujarat 380006, India; 4 Faculty of Critical Care Medicine, Wellstar Spalding Medical Center, Griffin, GA 30224, USA; 5 Faculty of Interventional Cardiology, Wellstar Spalding Medical Center, Griffin, GA 30224, USA; 6 Faculty of Interventional Cardiology, Cape Fear Valley Health, Fayetteville, NC 28304, USA

**Keywords:** electrical storm, ventricular tachycardia, implantable cardioverter-defibrillator, ischemic cardiomyopathy, antiarrhythmic therapy, catheter ablation

## Abstract

An electrical storm (ES) represents a critical arrhythmic emergency, often associated with high morbidity and mortality rates in patients with underlying structural heart disease. Despite therapeutic advancements, its management remains challenging. The present case report describes an exceptional case of ES marked by 42 appropriate implantable cardioverter-defibrillator (ICD) shocks within a period of 24 h, one of the highest documented burdens underscoring the limitations of conventional therapy and the importance of timely interventional strategies. A 71-year-old male patient with ischemic cardiomyopathy and prior MitraClip implantation presented with recurrent ICD shocks. Despite optimized device programming and dual antiarrhythmic therapy (amiodarone and lidocaine), he experienced 42 appropriate ICD discharges in a single day. His course rapidly progressed to severe hemodynamic instability, necessitating intubation, mechanical ventilation, deep sedation, and neuromuscular blockade. Telemetry demonstrated the transient suppression of ventricular tachycardia with pacing, followed in select episodes by degeneration into ventricular fibrillation, likely reflecting underlying substrate instability rather than device malfunction or direct pacing-induced proarrhythmia. This pattern suggested complex device-arrhythmia interactions and provided diagnostic insight. Following the failure of all conventional treatments, the patient was transferred for urgent electrophysiological intervention including catheter ablation and bilateral cardiac sympathetic denervation. Unfortunately, the patient did not survive. This case highlights an extreme and refractory form of ES in a patient with structural heart disease. The severity and resistance to medical therapy reinforce the need for early recognition and timely escalation to interventional approaches such as ablation and sympathetic denervation. Additionally, the captured telemetry offers unique electrophysiological insight into pacing-triggered ventricular arrhythmogenesis. The present case report contributes valuable clinical and diagnostic learning for physicians managing complex ventricular arrhythmias in advanced cardiac patients.

## Introduction

An electrical storm (ES) is a life-threatening cardiac emergency characterized by multiple episodes of sustained ventricular arrhythmias occurring within a 24-h time period. ES is commonly defined as ≥3 episodes of sustained ventricular tachycardia (VT)/ventricular fibrillation (VF) [or appropriate implantable cardioverter-defibrillator (ICD) therapies] in 24 h (1,2). In Western populations, ES occurs in ~4-28% of patients with ICDs, with higher rates observed in certain groups. Severe cases, particularly with hemodynamic instability or recurrent arrhythmias, carry an early risk of mortality >10% (2). VT storms are most frequently encountered in patients with structural heart disease and reduced ejection fraction (EF). However, they can also arise in individuals with structurally normal hearts due to diverse triggers, such as electrolyte disturbances, thyrotoxicosis, heightened sympathetic activity, or inherited cardiac conditions (3). ES management is complex, requiring a multidisciplinary approach that includes antiarrhythmic drugs, electrolyte correction and ICD therapy for optimal outcomes (4). Although refractory ES have been reported, few have demonstrated the extreme arrhythmic burden or intricate device-arrhythmia interactions seen here.

The present case report describes a rare, catastrophic case of VT storm in a patient with ischemic cardiomyopathy, marked by 42 ICD shocks in a period of 24 h, despite dual anti-arrhythmics and intensive support. The present case report also highlights the diagnostic and educational value of real-time telemetry and electrocardiogram (ECG) capture.

## Case report

The present case report describes a rare and life-threatening case of ES in a 71-year-old male patient with ischemic cardiomyopathy (EF, 35-40%), prior MitraClip placement with persistent moderate mitral regurgitation, cardiac resynchronization therapy defibrillator, multiple coronary stents and a history of pericardiocentesis to the Wellstar Spalding Medical Center (Griffin, GA, USA) on February 26, 2025. He presented with worsening exertional dyspnea, orthopnea, peripheral edema and non-radiating chest pressure, symptoms emerging after a recent reduction in diuretic therapy.

**Figure 1:**
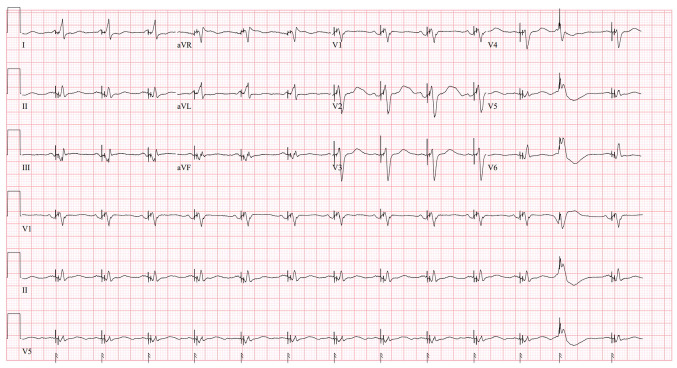
The 12-lead electrocardiogram demonstrates a ventricular paced rhythm, with an isolated premature ventricular complex occurring at the end of the tracing, superimposed on a pacemaker spike.

Upon admission, the patient was hypotensive (blood pressure, 90/70 mmHg) and tachycardic (hear rate, 130 beats per minute), with clinical features suggestive of evolving cardiogenic shock, including cool extremities, +3 bilateral lower extremity edema and a reduced urine output. Oxygen saturation remained 99% in room air. A laboratory evaluation demonstrated acute kidney injury, indicating early end-organ involvement. However, serum lactate levels were within normal range (1 mmol/l), suggesting compensated or early-stage shock physiology without overt systemic hypoperfusion at the time of initial evaluation. An initial 12-lead ECG (Fig. 1) demonstrated a ventricular paced rhythm with an isolated premature ventricular complex occurring at the end of the tracing, superimposed on a pacemaker spike, suggesting potential ectopic trigger. Laboratory analyses revealed a level of terminal pro-B-type natriuretic peptide of 16,800 pg/ml, an elevated creatinine level of 2.41 mg/dl (baseline, 1.80), and normal potassium (4.0 mmol/l), calcium (8.9 mg/dl), magnesium (2.0 mg/dl) and thyroid-stimulating hormone (2.2 µU/ml) levels, excluding common metabolic triggers.

Serum lactate levels were within normal range (1 mmol/l), suggesting adequate perfusion despite hypotension. The patient demonstrated renal dysfunction consistent with early end-organ involvement, while serum lactate levels remained normal, suggesting preserved systemic perfusion at the time of evaluation. The troponin level was mildly elevated at 102 ng/l, trending down to 90 ng/l, consistent with demand-mediated ischemia amid an ongoing ES. In the absence of dynamic ST changes and with prior coronary evaluation, acute coronary syndrome was considered unlikely, and no urgent angiography or stress testing was pursued. An echocardiography revealed a large inferobasal left ventricle/left ventricular (LV) aneurysm, severe left atrial enlargement, moderate mitral regurgitation, and no evidence of LV thrombus. Beta-blockers were avoided due to sustained hypotension and concern for exacerbating cardiogenic shock.

**Figure 2:**
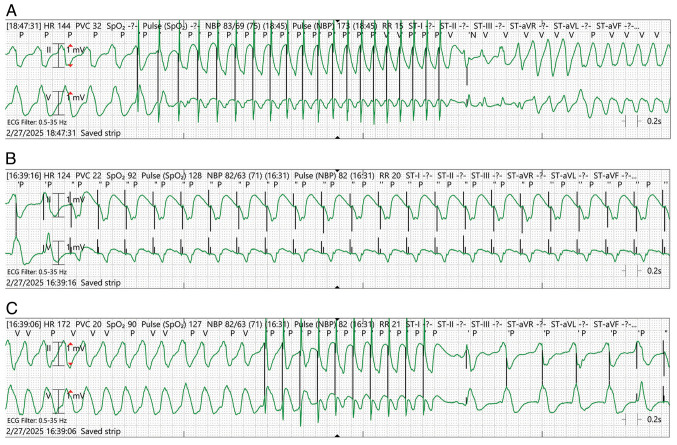
Telemetry tracings demonstrating device-arrhythmia interactions. (A) Initial VT terminated transiently by ramp pacing, followed by degeneration into ventricular fibrillation. (B) Sustained monomorphic VT with persistent ventricular pacing. (C) Burst pacing interrupts VT, followed by recurrence of slower VT. VT, ventricular tachycardia.

Shortly following his arrival, the patient developed sustained monomorphic VT, requiring ICD shocks. Despite intravenous amiodarone and lidocaine, he progressed to fulminant ES with 42 appropriate ICD shocks over a period of 24 h, along with two external cardioversions. Magnesium sulfate was administered empirically without benefit. Persistent VT prompted admission to the intensive care unit (ICU), intubation and initiation of deep sedation with neuromuscular blockade, though arrhythmias continued. Telemetry proved diagnostically valuable: As demonstrated in Fig. 2A, VT was transiently suppressed by overdrive pacing, followed in some episodes by degeneration into VF, suggesting a complex interaction between pacing therapy and an unstable arrhythmogenic substrate rather than inappropriate device function; As illustrated in Fig. 2B, sustained VT refractory to pacing was observed; and Fig. 2C illustrates VT interrupted by burst pacing, followed by slower recurrence. These recordings highlight complex device-arrhythmia interactions and the refractory nature of the storm.

ICD interrogation revealed optimal anti-tachycardia pacing and shock settings with no malfunctions; thus, reprogramming was not needed. The device documented a high VT burden with appropriate therapies delivered. The patient received a 150 mg intravenous amiodarone bolus followed by continuous infusion at 0.5 mg/min, and lidocaine was commenced at 1 mg/min after a 100 mg bolus. Despite dual antiarrhythmic therapy, inotropes and aggressive diuresis, VT persisted. Hemodynamics were tenuously maintained with norepinephrine. Mechanical circulatory support (e.g., intra-aortic balloon pump or Impella) was considered; however, these were unavailable at the treating center.

Urgent electrophysiology consultation recommended transfer for VT ablation and cardiac sympathetic denervation. However, due to regional ICU bed shortages, the patient remained at the referring facility. The prognosis remained poor. Despite transient stabilization with aggressive medical and supportive therapy, the clinical condition of the patient progressively deteriorated, and he unfortunately passed away on hospital day 5 from admission before advanced electrophysiologic therapies could be initiated.

## Discussion

ES may manifest as polymorphic VT, monomorphic VT, or VF, and is more commonly observed in patients with structural or infiltrative heart diseases, such as amyloidosis or sarcoidosis. Potentially reversible triggers include acute myocardial ischemia, electrolyte abnormalities, thyrotoxicosis, QT prolongation and drug toxicity. It may also be precipitated by reperfusion injury or myocardial scarring that serves as an arrhythmogenic substrate. Polymorphic VT is often associated with ischemia or metabolic disturbances, whereas monomorphic VT generally arises from scar-mediated reentry (2,5). The diverse pathophysiology of ES underscores the need to identify underlying triggers. In the case described herein, a large LV aneurysm, ischemic cardiomyopathy and prior pericardial intervention likely formed the substrate for the refractory monomorphic VT storm.

ES from VT commonly occurs in patients with structural heart disease, as in the case described in the present study. It can present with symptoms, such as palpitations, chest pain, dyspnea, or even cardiac arrest. Early resuscitation and ICU-level care are critical for stabilization and monitoring (1). The rapid progression from stable presentation to ICU intubation and multi-drug therapy over 24-48 h underscores the fulminant nature of electrical storm. The primary approach to treating patients with recurrent VT or VF relies on pharmacologic therapy, with antiarrhythmic agents such as amiodarone serving as a foundational component (6). Current guidelines support early beta-blocker use (e.g., IV esmolol or metoprolol) in ES. However, in the patient described herein, beta-blockade was deferred due to hypotension and suspected cardiogenic shock. Management followed guideline-based strategies, including dual antiarrhythmics, sedation to reduce sympathetic drive and vasopressors for hemodynamic support, aligning with the American College of Cardiology recommendations for aggressive, team-based ES care (2). Despite a continuous amiodarone infusion, the arrhythmia of the patient remained refractory, prompting the addition of lidocaine for enhanced rhythm control.

The observed transition from pacing-mediated VT suppression to subsequent VF likely reflects dynamic electrophysiologic substrate instability rather than pro-arrhythmic device behavior. In scar-mediated ventricular arrhythmias, pacing may transiently terminate re-entrant circuits, but can also alter conduction pathways, occasionally facilitating degeneration into more malignant rhythms in highly unstable myocardial substrates. Importantly, device interrogation in our patient confirmed appropriate sensing, detection and therapy delivery, supporting appropriate device function despite arrhythmia recurrence.

In ES, the prompt management of pain and psychological distress is critical, as recurrent external or ICD-mediated shocks can increase sympathetic activation through catecholamine surge, thereby exacerbating ventricular arrhythmias. Sedation with appropriate analgesia and anxiolysis is recommended to mitigate this adrenergic response and improve patient comfort. In the case described in the present study, deep sedation with neuromuscular blockade was implemented for sympathetic suppression and ventilator synchrony; however, the arrhythmia remained refractory. Current expert consensus supports sedation ranging from mild to deep levels for both arrhythmia control and patient comfort in ES management (2,7).

Percutaneous stellate ganglion block has emerged as a potential temporizing therapy for refractory ES by reducing sympathetic outflow and ventricular arrhythmia burden. Although this strategy has shown promising results as a bridge to definitive therapies (2), such as catheter ablation or surgical denervation, bedside SGB was not available at the authors' institution (Wellstar Saplding Medical Center) at the time of patient management and therefore, could not be pursued.

ES in patients with structural heart disease is associated with a high risk of early mortality, and catheter ablation, particularly when achieving complete VT elimination, provides a favorable outcome with ES-free survival rates >90% (8). Catheter ablation is a key therapy for refractory VT storm, particularly when complete VT suppression is possible. In the case in the present study, VT persisted despite dual antiarrhythmics, sedation and support, leading to plans for urgent ablation and sympathectomy. Unlike prior reports [e.g., Nakayama *et al* (8), Ahadzi *et al* (1) and Rahman and Sohail (4)], the patient in the present study experienced an unusually high ICD shock burden with no response to aggressive treatment.

In a previous study, a pooled meta-analysis of 471 patients with ES treated using catheter, ethanol, or surgical ablation reported favorable outcomes: In total, 72% achieved complete arrhythmia elimination, and 91% had successful suppression of clinical VTs (9). Complications were rare (2%), with <1% procedure-related mortality. At 1.2 years, 94% remained free of ES and 72% had no VT recurrence, though overall mortality was 17%, primarily due to progressive heart failure (9). In line with these findings, the patient in the present study was being prepared for transfer to a tertiary care center for consideration of VT ablation and cardiac sympathetic denervation, highlighting the critical role of advanced interventional strategies in refractory ES management.

Trans-coronary ethanol ablation has emerged as an option for refractory VT in structural heart disease, particularly when catheter ablation fails. In a series of 46 patients, selective coronary angiography guided ethanol delivery achieved partial success in 66%. However, VT has been shown to recur in 74% of patients at 6 months and 82% of patients at 12 months, with a 32% complication rate, including one procedural death (10).

Transcoronary ethanol ablation may benefit select patients; however, the patient described herein, with a large LV aneurysm and complex arrhythmogenic substrate, had not undergone ablation. Despite dual antiarrhythmics and supportive care, he experienced 42 ICD shocks in 24 h, highlighting a rare, severe VT storm and the urgent need for timely advanced electrophysiologic intervention in high-risk cases.

In conclusion, the present case report underscores the extreme and life-threatening nature of ES in patients with structural heart disease, particularly when refractory to optimized device settings and dual antiarrhythmic therapy. The documentation of 42 appropriate ICD shocks within 24 h represents one of the highest recorded burdens of therapy-resistant VT, highlighting the limitations of standard pharmacological and device-based management. The early recognition of ES physiology, combined with real-time diagnostic tools such as telemetry, is critical for timely intervention. The present case report reinforces the need for rapid multidisciplinary escalation, including electrophysiology consultation, catheter ablation and sympathetic denervation for improving outcomes in refractory cases. It also provides valuable electrophysiological insights into device-arrhythmia interactions that can inform future management of complex arrhythmias.

## Data Availability

The data generated in the present study may be requested from the corresponding author.
